# Malaysia: can ABHR purchasing data used to measure hand hygiene compliance?

**DOI:** 10.1186/2047-2994-4-S1-P296

**Published:** 2015-06-16

**Authors:** H Merican, YF Lee, R Nallusamy, LM Ong, P Mohamed Nazir, H Sham, N Ismail, M McLaws, Ministry of Health, Malaysia

**Affiliations:** 1 Penang State Health Department, Malaysia; 2 Infection Control Unit, Penang General Hospital, Malaysia; 3 Clinical Research Centre, Penang General Hospital, Malaysia; 4 Medical Development Division, Malaysia; 5 School of Public Health and Community Medicine, USNW Medicine, UNSW, Australia

## Introduction

After three years of mandatory quarterly hand hygiene audits in public Malaysian hospitals the burden of auditing is impacting the support and potential sustainability of the program. We looked to alternative methods to routinely measure compliance because human auditing has decreased validity and reliability inherent in the methodology.

## Objectives

Our objective was to test whether alcohol based hand rub (ABHR) purchase data could be used as a proxy for usage.

## Methods

All six public hospitals in Penang provided ABHR purchasing data between 2012 and 2014 and compliance rates that were collected from human auditing each quarter for the same period. Compliance rates were plotted against ABHR purchasing data adjusted by amount of solution required for a hand hygiene opportunity and tested for association after adjusting for a delay in usage after purchase.

## Results

Plotting the ABHR purchasing data against compliance rates illustrated a wide disparity between purchasing and human audits. When increases in compliance rates were examined for similar increases in ABHR usage, only small percentage point improves in compliance (1-3 percentage points) were accompanied by large increases in ABHR usage. Correlations, ranging from R^2^=0.0 to R^2^=0.1, demonstrated that there was no relationship between ABHR purchasing patterns and hand hygiene compliance at the six hospitals.

## Conclusion

No direct audit method that is ethically acceptable has both high validity and reliability and the Hawthorne effect from human auditing is difficult to quantify. Small increases in compliance were followed with incongruent large increases in ABHR purchase. The lack of correlation between ABHR and compliance rates suggests that compliance rates are not reflected in changes in ABHR purchasing patterns. The effect of adjusting ABHR purchasing data by patient days and other patient acuity data to improve the use of ABHR usage as a surveillance methodology for hand hygiene compliance will be discussed.

## Disclosure of interest

H. Merican: None declared, Y. F. Lee: None declared, R. Nallusamy: None declared, L. M. Ong: None declared, P. Mohamed Nazir: None declared, H. Sham: None declared, N. Ismail: None declared, M. McLaws Conflict with: MLM has previously been a WHO advisor and independent consultant on infection control surveillance to the Ministry. All authors declare no conflict of interest.

